# Modeling the Crack Interference in X80 Oil and Gas Pipeline Weld

**DOI:** 10.3390/ma16093330

**Published:** 2023-04-24

**Authors:** Wei Cui, Zhongmin Xiao, Qiang Zhang, Jie Yang, Ziming Feng

**Affiliations:** 1 School of Energy and Power Engineering, University of Shanghai for Science and Technology, Shanghai 200093, China; 2 School of Mechanical Science and Engineering, Northeast Petroleum University, Daqing 163318, China; 3 School of Mechanical and Aerospace Engineering, Nanyang Technological University, 50 Nanyang Avenue, Singapore 639798, Singapore; 4 School of Mechanical and Electrical Engineering, Wenzhou University, Wenzhou 325035, China

**Keywords:** unequal-length double cracks, virtual crack closure technique, crack interference, oil and gas pipeline weld, numerical modeling

## Abstract

Based on the numerical simulation method of the virtual crack closure technique (VCCT), an interference model was established to investigate the physical problem of two interacting cracks of different sizes in the welding zone of oil and gas pipelines. The obtained results of the current interference problem were compared with those of single crack case. Various crack configurations, such as different crack spacing and different crack sizes, were analyzed. The characteristic quantity fluid pressure load *P* during the crack propagation process, the peak value of the von Mises stress distribution field of the crack growth path, as well as the difference ∆*Bx* between the peak value of the magnetic induction intensity component at the crack and the value of the magnetic induction intensity component at its symmetrical position were calculated. The crack interaction scale factors, including $\gamma_{P}$, $\gamma_{\mathrm{Mises}}$, and $\gamma_{\Delta Bx}$, were compared and analyzed. The numerical modeling results show that when the unequal-length double cracks interfere with each other, the cracks are more likely to propagate toward each other. The tendency of the double-cracks to propagate toward each other gradually weakens as the distance between the crack tips increases and is finally the same as that of single-crack cases. It was also found that the effect of large-sized cracks on small-sized cracks is greater than that of small-sized ones on large-sized ones. The numerical modeling results could be applied for the prediction and analysis of multicrack damage in oil and gas pipeline welds.

## 1. Introduction

Oil and natural gas are dangerous goods under national key supervision due to their flammable, explosive, and toxic characteristics. Large storage tanks and oil and gas pipelines used to store and transport oil and gas resources easily constitute a major hazard. Under the combined action of internal and external loads, this storage and transportation equipment will inevitably produce defects and cracks, especially in stress-concentration areas such as welds. Leakage and explosion accidents of storage and transportation equipment frequently occur owing to weld defects. For example, in 1974, an accident occurred at the Mitsubishi Mizushima Oil Refinery in Japan. The weld between the oil tank wall and the side plate of the tank bottom that was 5 × 10^4^ cubic meters fractured, and the oil leaked out instantaneously and destroyed the fire embankment, posing a serious threat to the environment and the safety of people’s lives and property. The southwest oil and gas field’s “1.20” explosion and fire accident is another example. The direct cause of the accident was that the pipe was torn under internal pressure due to defects in the spiral weld of the pipe, and the ferrous sulfide powder carried by the leaked natural gas oxidized and spontaneously ignited in the air, causing an explosion outside the natural gas pipe. On 22 November 2013, a particularly serious accident occurred in Qingdao, Shandong Province, resulting in 62 deaths and a direct economic loss of CNY 751.72 million. In the analysis of this accident, “Safety Culture Network” showed that weld defects and corrosion are the two main internal reasons for the leakage of old pipelines in service. Therefore, the safety management, detection, and protection of storage and transportation equipment are the key issues related to the prevention and control of safety production accidents. The fractures of storage tank steel and pipeline steel are generally ductile fractures. Once ductile fractures occur, the cracks will gradually propagate and are difficult to stop until the above-mentioned catastrophic accidents are caused [1,2,3,4,5,6]. Cracks in actual pipelines mostly exist in the form of crack groups. When multiple cracks coexist, the mutual interference between cracks is more likely to cause safety accidents. Therefore, the propagation law of multicrack interference has become a hot research issue.

Researchers have conducted relatively in-depth analyses on crack growth in term of theoretical research, numerical simulation, and experiment. Jin and Wu performed fatigue loading tests on 2060 Al-Li alloy specimens that contain single and multiple cracks. Experimental results revealed that when there is no interaction between the multiple cracks, the fatigue crack growth rate deviates from that of a single crack. For collinear cracks of equal and unequal crack sizes, all the da/dN vs. $\Delta K_{n}$ curves overlap with those single-crack curves (without crack interaction). This indicates that $\Delta K_{n}$ can be seen as a suitable parameter to handle the fatigue behavior of materials containing multiple cracks [7]. Using a phase-shifted coherent gradient sensing method, Ma and Xie conducted the mixed-mode fracture investigation of polymethyl methacrylate (PMMA) for initial single/double crack(s) interference. To depict the fracture around the crack tip in various ways, such as fracture toughness, fracture mixing, stress intensity factor, crack initiation, and propagation, a series of coherent gradient sensing (CGS) interferograms were employed. The impact of offset distance on fracture characterization was discussed, and the interference impact of double cracks was investigated along with multiple crack lengths and distances. Good agreement is shown in comparing the numerical results with experimental K-dominance results [8]. Galatolo and Lazzeri tackled the fatigue propagation of multiple cracks in panels with holes of finite width which are typical aircraft structural components. In their study, fatigue testing campaigns were performed on six different settings of notches and cracks, which include several interacting cracks around the holes. By comparison of the experimental results with those from the implemented models, a good agreement can be obtained [9]. Xu et al. investigated a linearly independent higher-order extending numerical manifold method and its application to multiple crack growth simulations. They found that the requirements of fracture mechanics and mechanical balance can be met by the trial-and-error method and the alteration of load multiple in the process of crack growth. The propagation paths of multiple cracks can be accurately predicted by the proposed extended linear independent numerical manifold method (NMM) [10]. Fageehi and Alshoaibi used fracture and crack growth analysis to deal with a two-dimentional finite element simulation of nonplanar multiple cracks. For multiple-crack cases, the cracks propagate sequentially according to the magnitude of the stress intensity factor, and the direction of crack propagation is also affected by the existence of other cracks, which attract each other depending on the position of one relative to another [11]. Jin et al. investigated the fatigue growth behavior of two interacting cracks with various crack offsets. The results note that the interaction will change from positive to negative in the process of multicrack growth, resulting in faster or slower crack growth rate, indicating that the impact of interaction on crack growth behavior changes with the different phases of crack growth [12]. On crack growth paths and associated stress intensity factors (SIFs), a numerical simulation was performed by Alshoaibi and Fageehi for linear elastic materials. Depending on the location of the hole, the results show that the crack grows along the direction of the hole because of the uneven stress at the crack tip, which is the result of the influence of the hole. The model was validated in terms of crack propagation trajectories and SIFs in several crack propagation studies reported in the literature [13]. The robust decision of fatigue crack growth thresholds from crack propagation data was investigated by Schonherr et al. The results show that the proposed method decreases the artificial conservatism led by the evaluation method as well as the sensitivity of test data dispersion and the impact of test data density [14]. Zhang and Collette investigated the use of dynamic Bayesian networks to predict the propagation and interaction of multicracks in structural systems. Using the crack lengths measured in the experiment, the ability of predicting future crack size evolution is assessed. For this sample, it must capture a high level of crack-to-crack interaction so that the pair of cracks can realistically track the evolution of the system [15]. Ahmed et al. used the boundary cracklet method to study the fatigue crack propagation of multiple interacting cracks in a porous perforated plate. The boundary cracklet method (BCM) was further used to model the interaction of multiple fatigue cracks in a perforated plate geometry with multiple holes and cracks. They concluded that the BCM could be a reliable tool for simulating the reality of multiple fatigue crack interactions in 2D [16].

Among the finite element methods for studying crack growth, the virtual crack closure technique (VCCT) is one of the most widely used methods [17,18,19,20,21,22,23]. For example, Krueger outlined a few virtual crack closure techniques. The methodology used was discussed, the history was summarized, and insights into its application were provided. Necessary modifications to the use of geometrically nonlinear finite element analysis methods and corrections required for crack-tip elements of different lengths and widths were analyzed [17]. Banks-sills and Farkash studied the specification of the virtual crack closure technique for a crack at a bimaterial interface. The energy release rate’s dependence on the virtual crack growth size of the interfacial crack was analyzed and accounted for so that the stress intensity factor can be obtained accurately when using a fine finite element mesh, as well as by more than one element [18]. Yu et al. studied the crack growth behavior of simulated nuclear graphite using the extended finite element method (XFEM), VCCT, and cohesive zone model (CZM) methods. The dependence on the element size that the peak load *P_c_* has was analyzed, and the numerical results showed that, using the three methods, *P_c_* is sensitive to the mesh size, and most sensitive when using VCCT [19]. Yao et al. investigated a 3D-VCCT-based fracture analysis method for multicracked gas pipelines. If the crack horizontal spacing is greater than six times the major semiaxis of the main crack, the interference between parallel collinear cracks and parallel offset cracks is negligible, and the analysis of multiple cracks can be simplified to a single crack in the process [20]. Based on the previous research work of the research group [24,25], this paper carried out the research on the interference problem of unequal-length cracks in the weld seam of X80 oil and gas pipeline, and the research results can provide theoretical basis for guiding the actual safety assessment of X80 pipeline with multiple cracks.

## 2. Unequal-Length Cracks Interference Method

**Figure 1:**
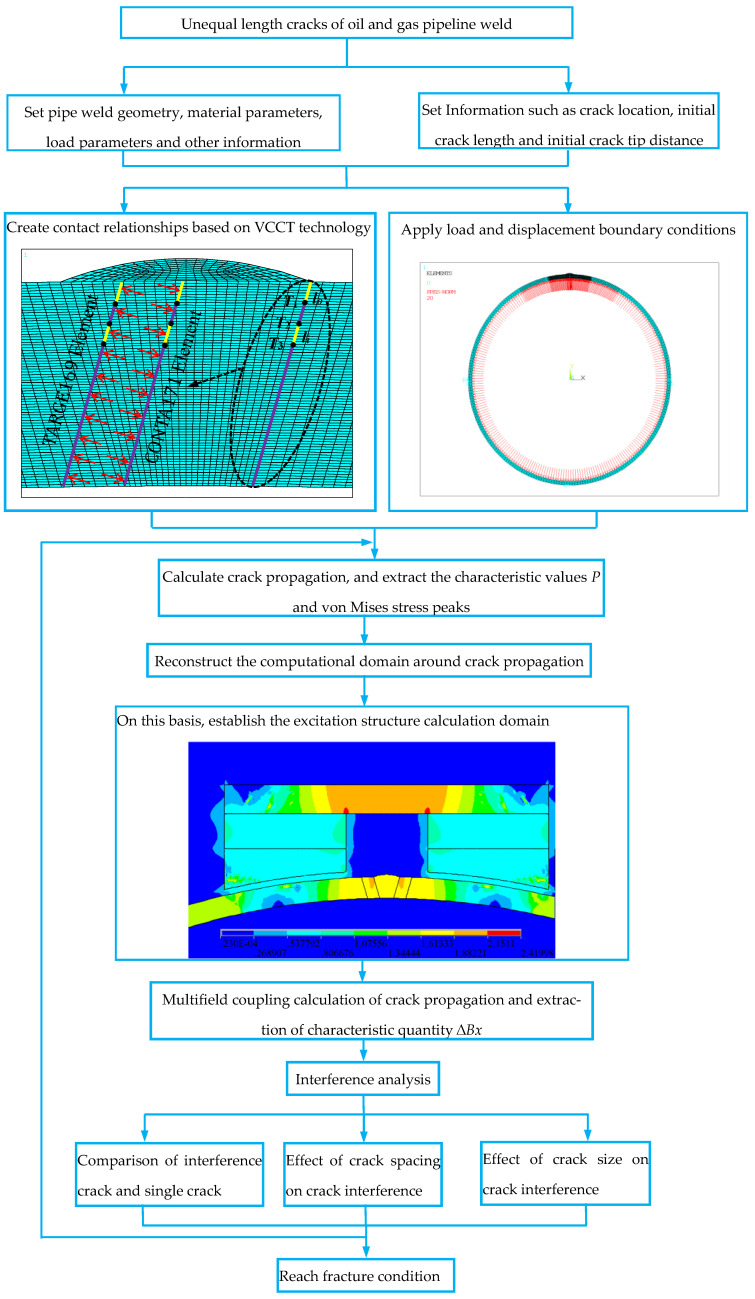
Flow chart of interference method for unequal-length cracks.

**Figure 2:**
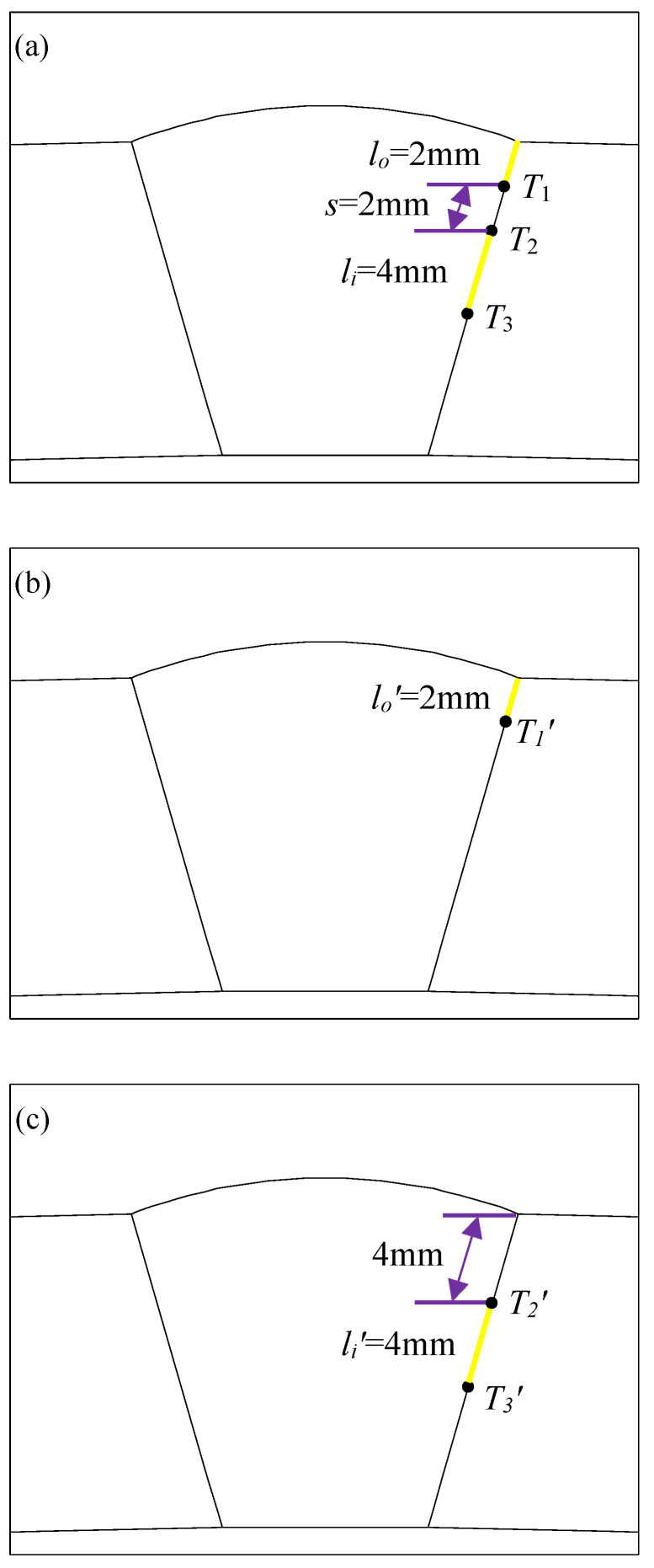
Crack model of pipeline weld: (**a**) model of double cracks with unequal length of the pipeline weld; (**b**) model of single crack in the outer wall of the pipeline weld; (**c**) model of single crack in the internal of the pipeline weld.

**Figure 3:**
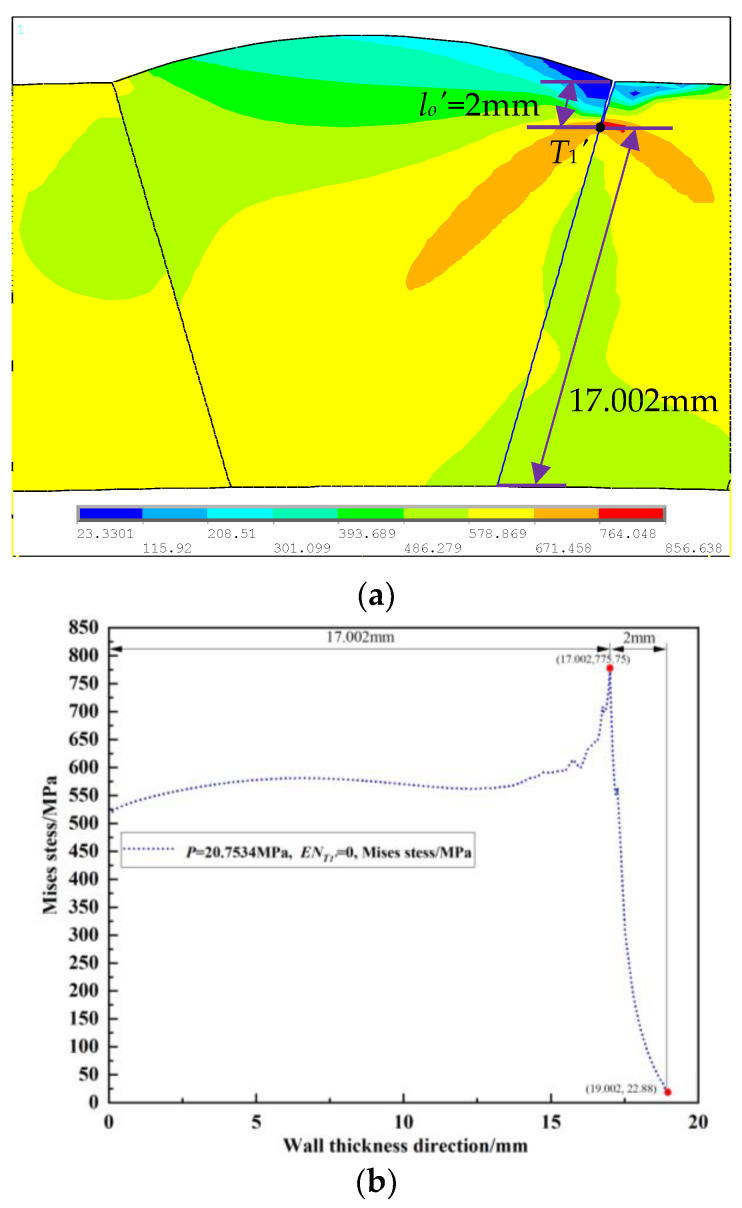
Single crack in outer wall: (**a**) von Mises stress distribution nephogram; (**b**) von Mises stress distribution field in the crack propagation path.

The flow chart of the established unequal length crack interference method is shown in Figure 1. Step 1: Establish the unequal-length crack model of the X80 oil and gas pipeline weld, and set the geometric dimensions, material parameters, load parameters, and other information of the pipeline weld. Geometric dimensions and material parameters are shown in Table 1 and Figures 2 and 3 in the previous study [24] and Tables 1 and 2 in the previous study [25]. The initial fluid pressure in the pipeline is 1 MPa; the maximum fluid pressure is 30 MPa. The model properties are shown in Table 1 of the paper. Step 2: Set information such as the position of the unequal-length crack, the initial length of the crack, and the initial distance of the crack tip. Step 3: Based on VCCT technology; discretize the prepropagation path into INTER202 interface units; select TARGE169 as the target unit at the interface unit; select CONTA171 as the contact unit; and create a contact relationship. Step 4: Apply load and displacement boundary conditions; constrain the degrees of freedom in the x-direction and y-direction at 0° and 180° in the circumferential direction of the pipeline; constrain the degree of freedom in the x-direction at 270° in the circumferential direction of the pipeline. Step 5: Based on the energy release rate criterion, carry out the unequal-length crack propagation calculation and extract the first characteristic quantity during crack propagation process, the fluid pressure load *P* in the pipeline, and the second characteristic quantity, the von Mises stress distribution field peak value, of the crack propagation path. Step 6: Reconstruct the calculation domain around the unequal length crack growth. The specific implementation process is to extract the propagation result of each load step during the crack propagation process, update the node coordinates according to the deformation during the process, and perform mesh reconstruction around cracks with unequal lengths. Step 7: According to the structural characteristics of the pipeline weld, construct the calculation domain of the pipeline external excitation structure of the permanent magnet, armature, and pole shoe. Step 8: With the dynamic application of the fluid pressure, the crack initiation pressure is reached, and the crack is initiated. The fluid pressure continues to be dynamically increased, which accelerates the crack propagation process, causes the deformation of the pipeline weld structure, and leads to the change of the leakage magnetic field distribution. In the multifield coupling calculation process, the difference ∆*Bx* between the peak value of the magnetic induction intensity component at the crack and the value of its symmetrical position of the third characteristic quantity in the process of crack propagation is extracted. In the process of unequal-length crack propagation, the incremental crack propagation of each load step is completed, and the excitation structure model is established based on the reconstructed mesh. The loop iteratively calculates the multifield coupling of crack propagation until the fracture condition is reached.

## 3. Simulation Study of Unequal-Length Cracks Interference

Based on the unequal-length crack interference method above, the simulation research of unequal-length crack interference was carried out, and the characteristic quantity fluid pressure load *P* in the process of crack propagation, the peak value of von Mises stress distribution field in the crack propagation path, the difference value ∆*Bx* between the peak value of magnetic induction component at the crack, and the value at its symmetric position were compared and analyzed. On this basis, the crack interaction ratio factor was introduced to study the influence of crack spacing on crack interference and crack size on crack interference.

### 3.1. Interference Phenomenon of Unequal Length Cracks

For the problem of ferromagnetic pipeline welds with double cracks, in order to study the interference effect of unequal-length double cracks, a model of unequal-length double cracks in pipeline welds was established, as shown in Figure 2a. The double crack consists of a crack in the outer wall of the pipeline weld and a crack in the inside of the pipeline weld. In order to compare the interference effect, a single crack model on the outer wall of the pipeline weld was established at the same position, as shown in Figure 2b, and another single crack model inside the pipeline weld was established, as shown in Figure 2c. Three models of cracks are set in Figure 2, with the yellow lines representing cracks. The two unequal-length cracks in Figure 2a are located at the same position and have the same length as the single cracks in Figure 2b,c, that is, the initial length of the outer wall crack is *l_o_* = *l_o_*′ = 2 mm, and the initial length of the internal crack is *l_i_* = *l_i_*′ = 4 mm, and the initial distance between the crack tips of two unequal-length cracks is *s* = 2 mm. In Figure 2a, the crack tips are indicated by *T*_1_, *T*_2_, and *T*_3_; in Figure 2b, the crack tips are indicated by *T*_1_′; and in Figure 2c, the crack tips are indicated by *T*_2_′ and *T*_3_′. *EN* represents the finite element, *EN_T_*_1_ represents the number of finite elements extended by the crack tip *T*_1_, and so on. When meshing, the discrete size of the unit on the crack propagation path was 0.25 mm.

**Figure 4:**
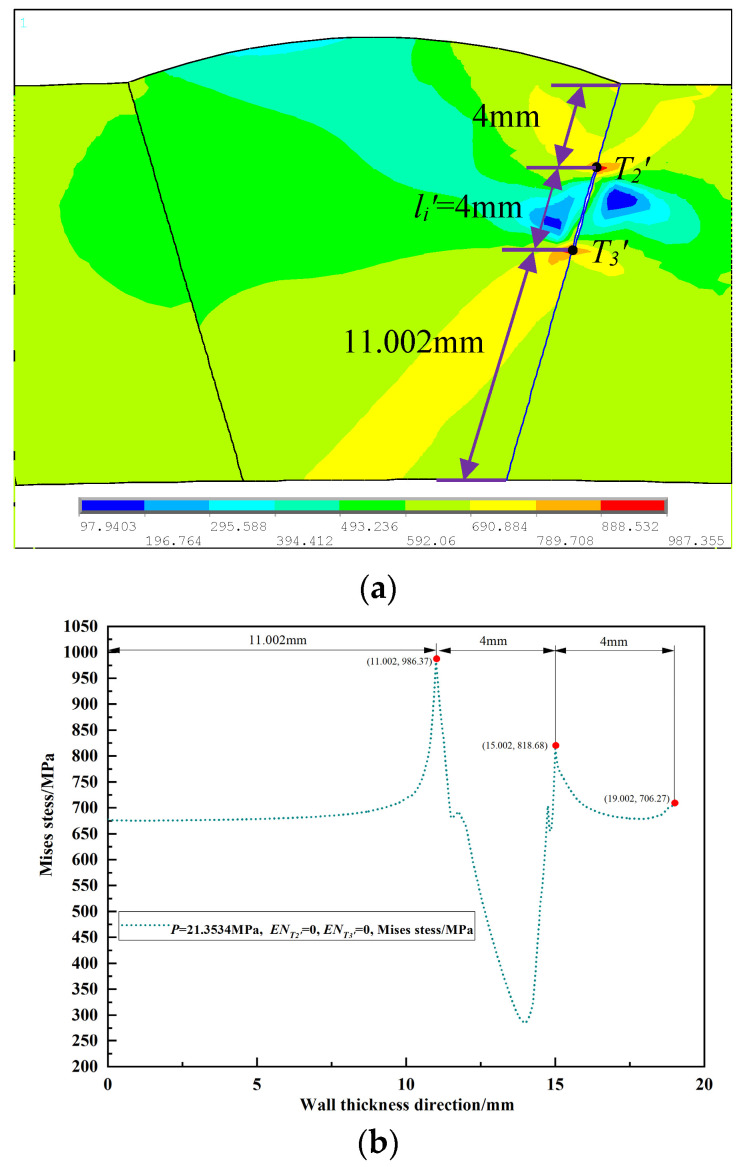
Internal single crack: (**a**) von Mises stress distribution nephogram; (**b**) von Mises stress distribution field in the crack propagation path.

**Figure 5:**
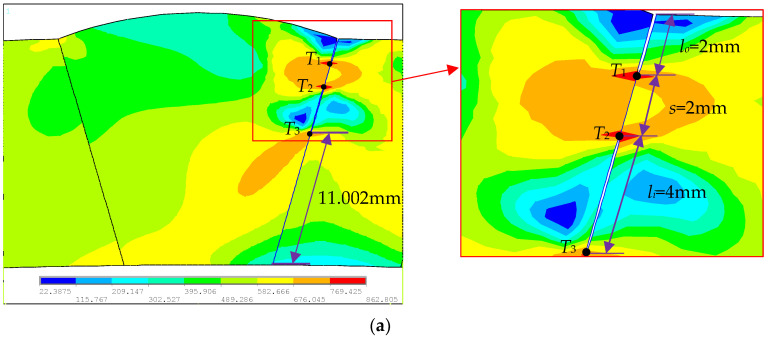

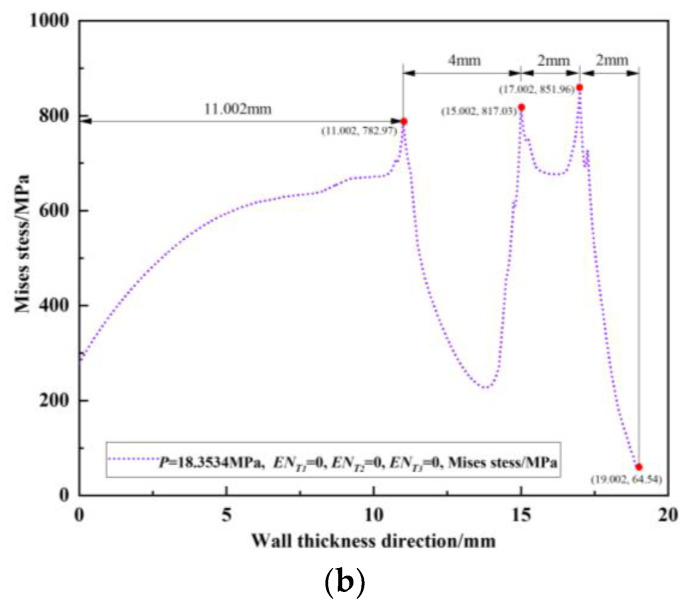
Unequal-length double cracks: (**a**) von Mises stress distribution nephogram; (**b**) von Mises stress distribution field in the crack propagation path.

The von Mises stress during the propagation process of the outer wall single crack (*p* = 20.7534 MPa), the internal single crack (*p* = 21.3534 MPa), and the unequal-length double cracks (*p* = 18.3534 MPa) in the loading step before the crack propagation were extracted. Their distribution cloud maps are shown in Figure 3a, Figure 4a and Figure 5a, and the von Mises stress distribution fields of their corresponding crack propagation path are shown in Figure 3b, Figure 4b and Figure 5b. It can be seen from Figure 3 that the peak value of the von Mises stress distribution field of the crack propagation path is located at 2 mm from the outer wall, which is at the crack tip *T*_1_′ of the single crack on the outer wall. Additionally, it can be seen from Figure 4 that the two peaks of the von Mises stress distribution field of the crack propagation path are located at 4 mm and 8 mm from the outer wall, which are the crack tips *T*_2_′ and *T*_3_′ of the internal single crack. In Figure 5, it appears that the three peaks of the von Mises stress distribution field of the crack propagation path are located at 2 mm, 4 mm, and 8 mm from the outer wall, which are the double crack tips *T*_1_, *T*_2_, and *T*_3_. From Figure 3, Figure 4 and Figure 5, it can be seen that the place where the von Mises stress is the largest is at the crack tip; the distance between the peak value of the von Mises stress distribution field and the internal or outer wall of the pipeline is consistent with the distance from the crack tip to the internal or outer wall of the pipeline. Therefore, the position of the single crack or double crack tips can be judged from the peak position of the von Mises stress distribution field.

**Figure 6:**
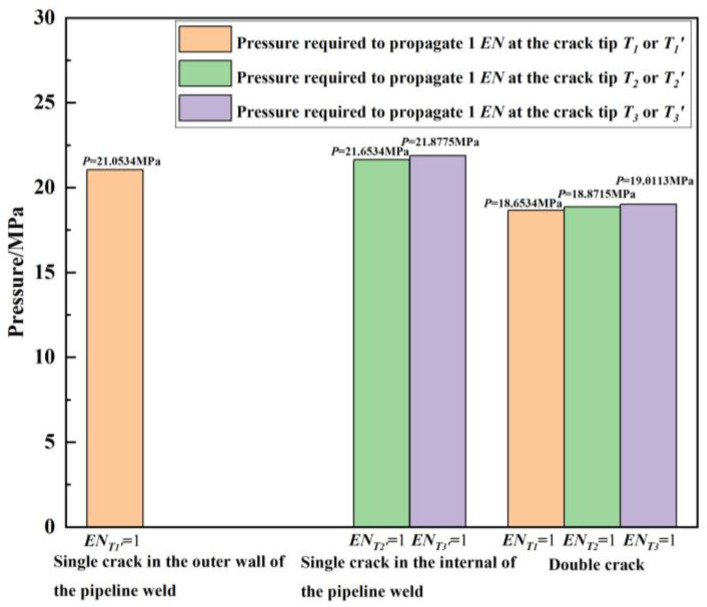
Pressure load required for the crack tips corresponding to the outer wall single crack, internal single crack, and double cracks to extend by 1 *EN*, respectively.

Figure 6 shows the required pressure load for the crack tips corresponding to the outer wall single crack, internal single crack, and double crack to extend by one finite element (*EN*), respectively. It can be seen from the figure that due to the interference effect of the internal crack, the pressure load required for the outer wall crack tip *T*_1_ to extend by 1 *EN* in the double cracks is reduced by 21.0534 − 18.6534 = 2.4 MPa compared to the pressure load required for the outer wall single crack tip *T*_1_′ to extend by 1 *EN*. Similarly, due to the interference effect of the outer crack, the pressure load required for the internal crack tip *T*_2_ to extend by 1 *EN* is reduced by 21.6534 − 18.8715 = 2.7819 MPa compared to the pressure load required for the internal single crack tip *T*_2_′ to extend by 1 *EN*. Additionally, the pressure load required for the crack tip *T*_3_ to extend by 1 *EN* in the double cracks is reduced by 21.8775 − 19.0113 = 2.8662 MPa compared to the pressure load required for the internal single crack tip *T*_3_′ to extend by 1 *EN*. Judging from the pressure load *P* required for crack propagation, the interference effect between the double cracks intensifies the crack propagation process compared with a single-crack case.

**Figure 7:**
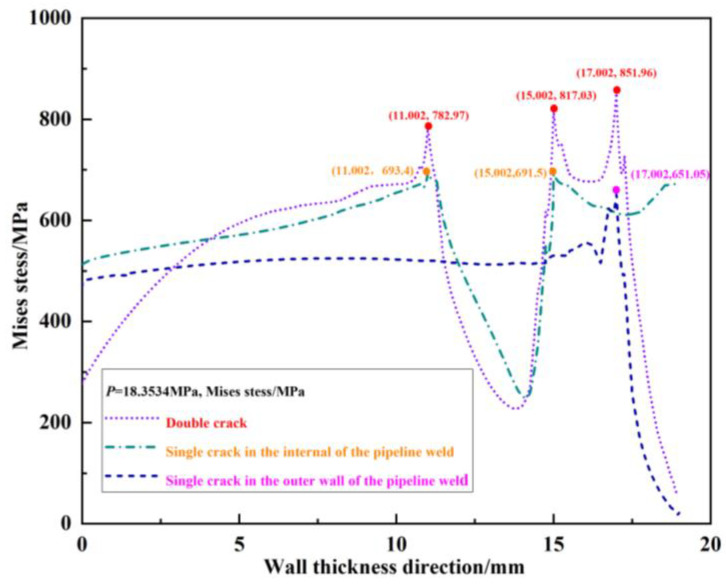
Von Mises stress distribution field of the crack propagation path when the outer wall single crack, internal single crack, and double crack are under the same pressure load.

The von Mises stress distribution field of the crack propagation paths for the outer wall single crack, internal single crack, and unequal-length double cracks under the same pressure load *p* = 18.3534 MPa without any propagation is shown in Figure 7. It can be seen from the figure that under the same pressure load *p* = 18.3534 MPa, due to the interference effect of the internal crack, the peak von Mises stress at the outer wall crack tip *T*_1_ of the double cracks increases by 851.96 − 651.05 = 200.91 MPa compared to the peak von Mises stress at the outer wall single crack tip *T*_1_′. Because of the interference effect of the outer wall crack on the internal crack, the von Mises stress peak value of the crack tip *T*_2_ in the double cracks increases by 817.03 − 691.5 = 125.53 MPa compared to the peak von Mises stress at the internal single crack tip *T*_2_′. The peak von Mises stress at the crack tip *T*_3_ increases by 782.97–693.4 = 89.57 MPa compared to the peak von Mises stress at the internal single crack tip *T*_3_′. Judging from the von Mises stress at the crack tip, compared with the single crack, the interference effect between the double cracks intensifies the crack propagation process.

**Figure 8:**
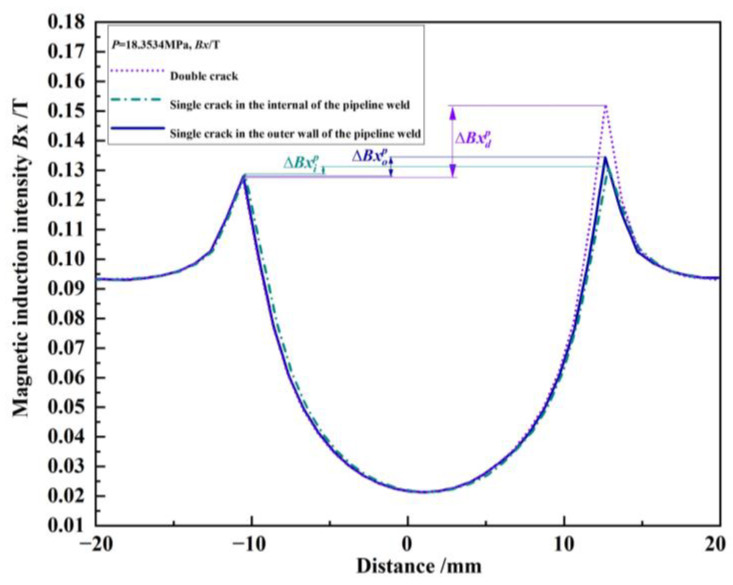
Magnetic induction intensity component curves of the outer wall single crack, internal single crack, and double crack under the same pressure load.

The component curves of magnetic induction intensity for the outer wall single crack, internal single crack, and unequal-length double cracks under the same pressure load *p* = 18.3534 MPa without any propagation were extracted and are shown in Figure 8. Since the numerical model only establishes the relevant cracks on the right side, and the previous research results show that the magnetic induction intensity curve is symmetrical when there is no crack in the pipeline weld, the characteristic quantity ∆*Bx* is defined as the difference between the peak value of the magnetic induction intensity component at the crack and its symmetrical position [24,25]. In this study, there are, in total, three ∆*Bx* characteristic quantities, with ∆*Bx_o_^p^* representing the outer wall single crack, ∆*Bx_i_^p^* for the internal single crack, and ∆*Bx_d_^p^* for the double cracks. In Figure 8, ∆*Bx_o_^p^* = 0.0065 T, ∆*Bx_i_^p^* = 0.0025 T, ∆*Bx_d_^p^* = 0.0246 T. The interference effect between the double cracks leads to the superposition of the leakage magnetic field of the outer wall crack with that of the internal crack. Furthermore, it leads to the detected peak value of the magnetic induction intensity component of the double cracks increasing by ∆*Bx_d_^p^* − ∆*Bx_o_^p^* = 0.0181 T compared to the peak value of the magnetic induction intensity component of the single crack in the outer wall. So, the detected peak value of the magnetic induction intensity component of the double cracks increases by ∆*Bx_d_^p^* − ∆*Bx_i_^p^* = 0.0221 T compared to the peak value of the magnetic induction intensity component of the internal single crack. Judging from the peak value of the magnetic induction intensity component, compared with the single crack, the interference effect between the double cracks intensifies the crack propagation process. It can be seen that when the double cracks interfere with each other, the cracks are more likely to propagate toward each other.

**Figure 9:**
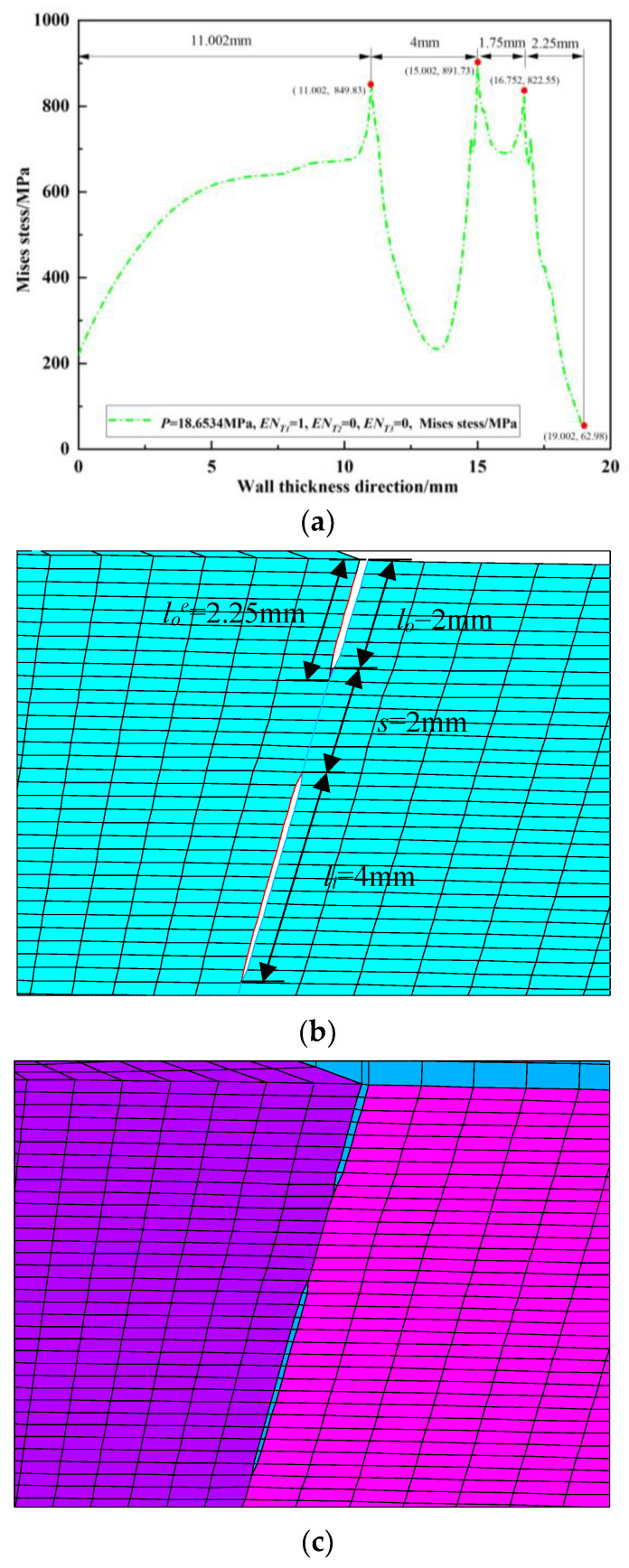
Crack tip *T*_1_ extending by 1 finite element: (**a**) von Mises stress distribution field; (**b**) finite element model in the extension process; (**c**) grid reconstruction.

**Figure 10:**
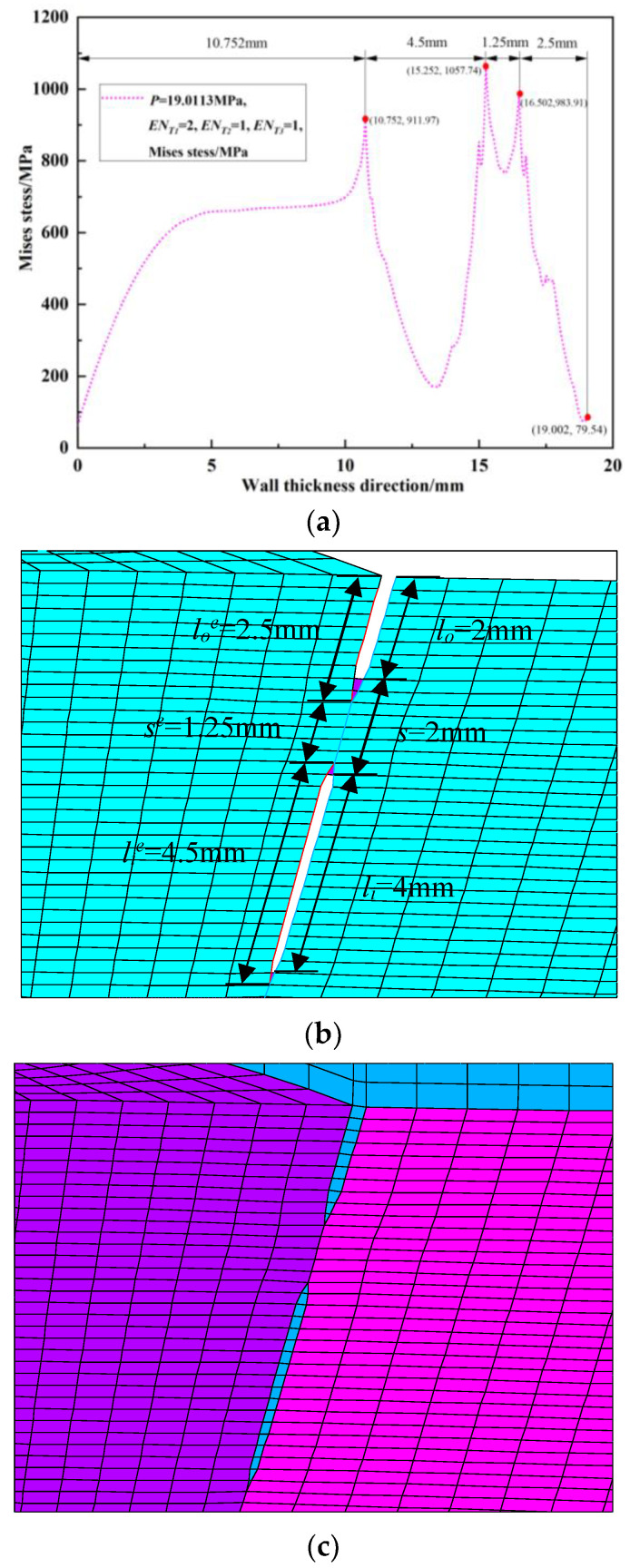
Crack tip *T*_1_ extending by 2 finite elements: (**a**) von Mises stress distribution field; (**b**) finite element model in the extension process; (**c**) grid reconstruction.

**Figure 11:**
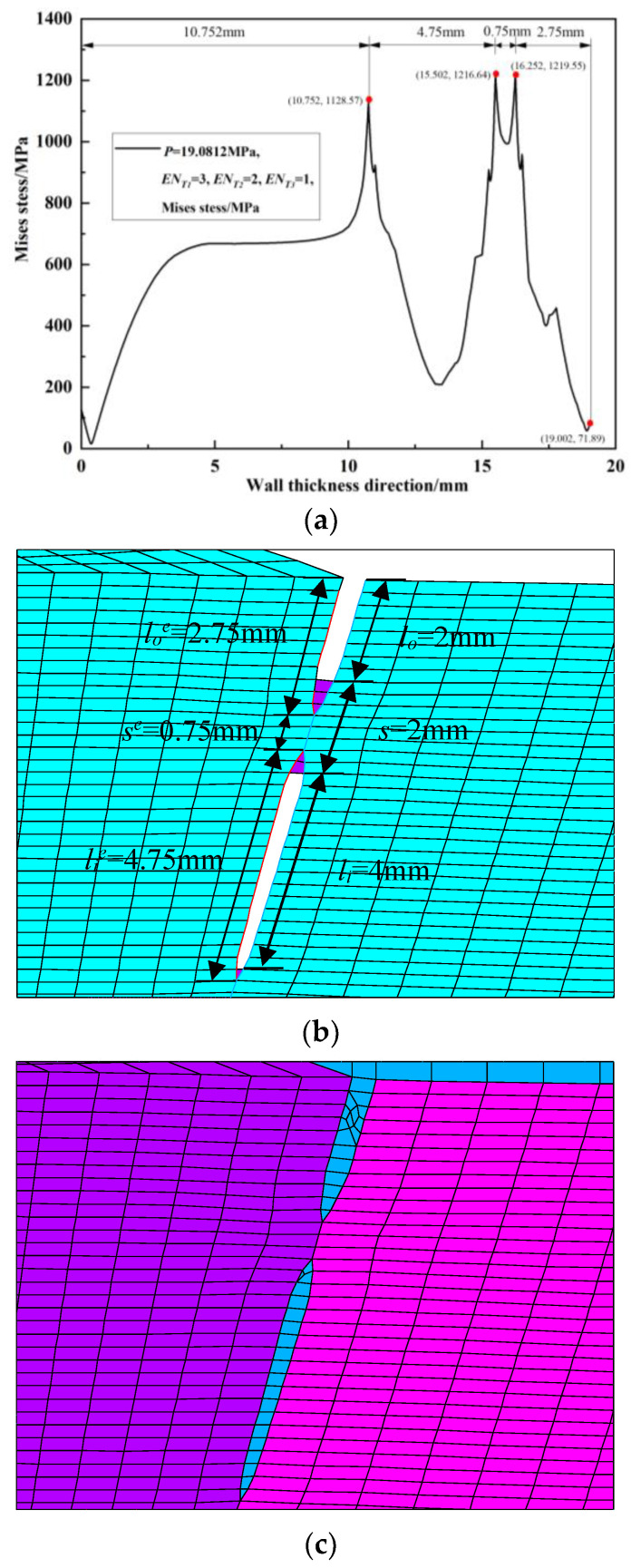
Crack tip *T*_1_ extending by 3 finite elements: (**a**) von Mises stress distribution field; (**b**) finite element model in the extension process; (**c**) grid reconstruction.

**Figure 12:**
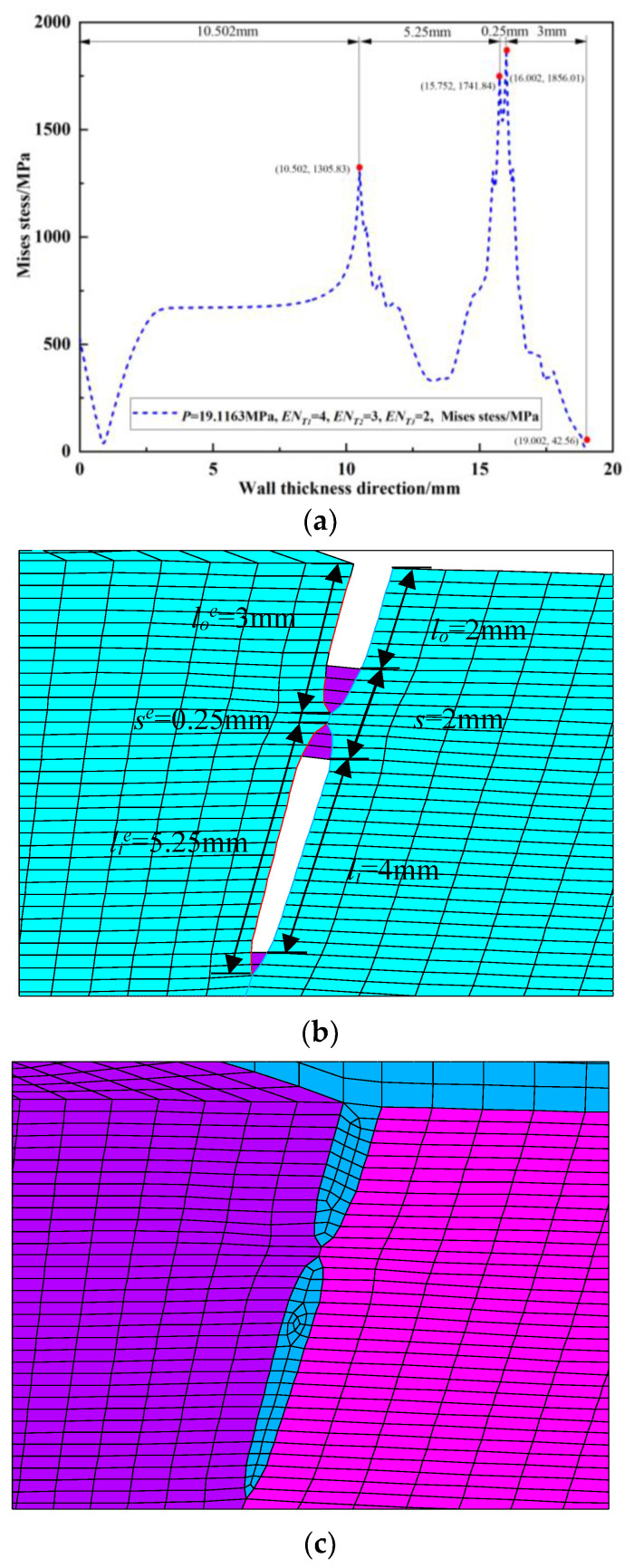
Crack tip *T*_1_ extending by 4 finite elements: (**a**) von Mises stress distribution field; (**b**) finite element model in the extension process; (**c**) grid reconstruction.

**Figure 13:**
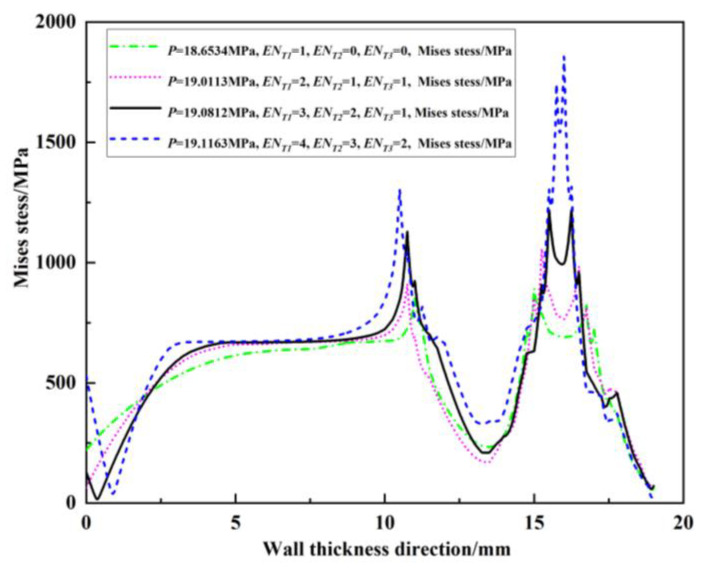
Summary of von Mises stress distribution fields when *EN_T_*_1_ = 1, *EN_T_*_1_ = 2, *EN_T_*_1_ = 3, *EN_T_*_1_ = 4.

**Figure 14:**
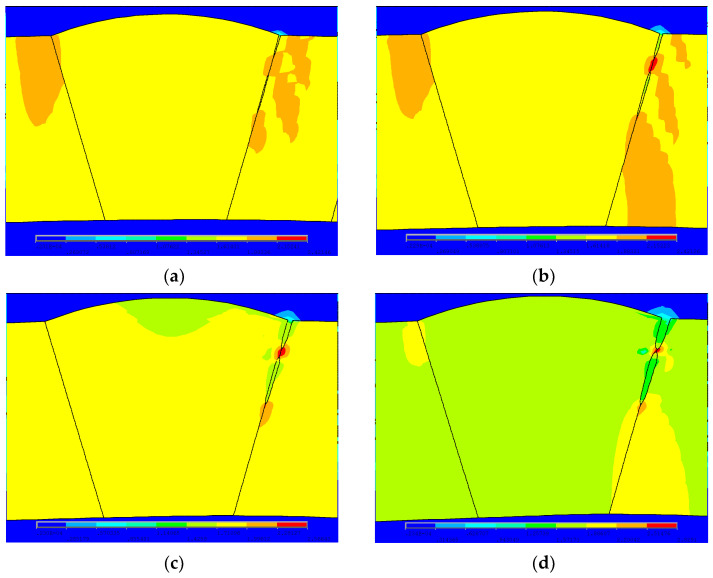

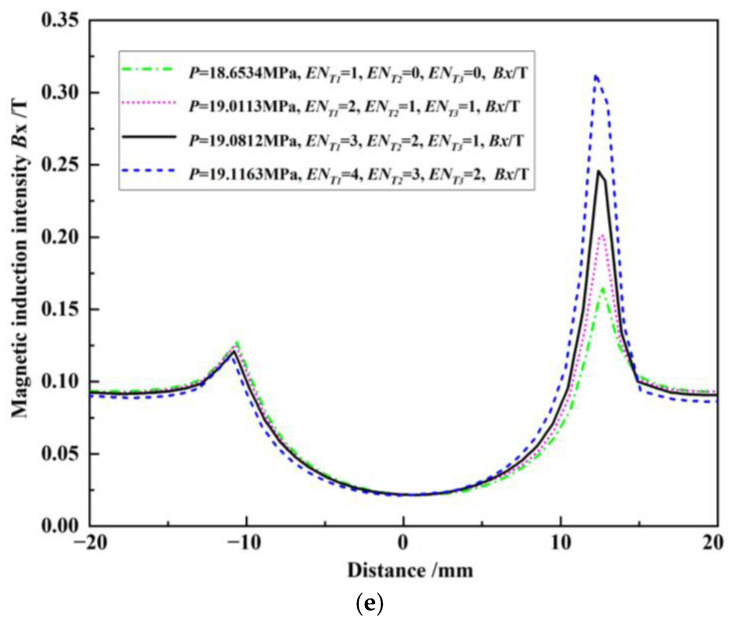
Magnetic induction intensity nephograms and component curves of magnetic induction when *EN_T_*_1_ = 1, *EN_T_*_1_ = 2, *EN_T_*_1_ = 3, *EN_T_*_1_ = 4: (**a**) magnetic induction intensity nephogram at the crack when *EN_T_*_1_ = 1. (**b**) magnetic induction intensity nephogram at the crack when *EN_T_*_1_ = 2. (**c**) magnetic induction intensity nephogram at the crack when *EN_T_*_1_ = 3. (**d**) magnetic induction intensity nephogram at the crack when *EN_T_*_1_ = 4. (**e**) component curves of magnetic induction when *EN_T_*_1_ = 1, *EN_T_*_1_ = 2, *EN_T_*_1_ = 3, *EN_T_*_1_ = 4.

Since the peak position of the von Mises stress distribution field is at the crack tip, the position and number of cracks can be judged. Extracting *s* = 2 mm during the crack propagation process of the double crack in the Figure 2a above, the von Mises stress distribution field of the crack tip *T*_1_ extending by one, two, three, and four finite elements, that is, the von Mises stress distribution fields of *EN_T_*_1_ = 1, *EN_T_*_1_ = 2, *EN_T_*_1_ = 3, and *EN_T_*_1_ = 4, are depicted in Figure 9a, Figure 10a, Figure 11a and Figure 12a. In Figure 9b, Figure 10b, Figure 11b and Figure 12b, the length of the outer wall crack is expressed by *l_o_^e^*, and the length of the internal crack is expressed by *l_i_^e^*. The distance between the crack tip *T*_1_ and the crack tip *T*_2_ is denoted by *s^e^*. Grid reconstructions are shown in Figure 9c, Figure 10c, Figure 11c and Figure 12c. It is seen from Figure 9, Figure 10, Figure 11 and Figure 12 that as the crack propagates, the peak position of the von Mises stress distribution field also changes, and there is a one-to-one correspondence. For example, in Figure 12b, when *EN_T_*_1_ = 4 and *EN_T_*_2_ = 3, the distance between the crack tip *T*_1_ and the crack tip *T*_2_ is a finite element, *s^e^* = 0.25 mm, while in Figure 12a, the corresponding peak position coordinates of the von Mises stress distribution field at this time are (15.752, 1741.84) and (16.002, 1856.01), and the distance between the two peaks is 16.002–15.752 = 0.25 mm. The von Mises stress distribution fields of *EN_T_*_1_ = 1, *EN_T_*_1_ = 2, *EN_T_*_1_ = 3, and *EN_T_*_1_ = 4 are summarized in Figure 13. This figure shows that when the crack tip begins to propagate, the von Mises stress value at the crack tip increases as the crack propagation process intensifies. From Figure 13, the crack propagation process and position can be monitored more clearly. Figure 14a–d show the magnetic induction intensity nephograms when extracting crack tip *T*_1_ extending by one, two, three, and four finite elements, and Figure 14e shows the magnetic induction intensity component curves when extracting crack tip *T*_1_ extending by one, two, three, and four finite elements. With the increase in the crack propagation process, the greater the peak value of the von Mises stress at the crack tip, the greater the deformation at the crack position, which affects the magnetic field distribution of the pipeline weld during the crack propagation process, so the peak value of the detected magnetic induction intensity component shows an increasing trend.

### 3.2. Effect of Crack Spacing on Crack Interference

**Figure 15:**
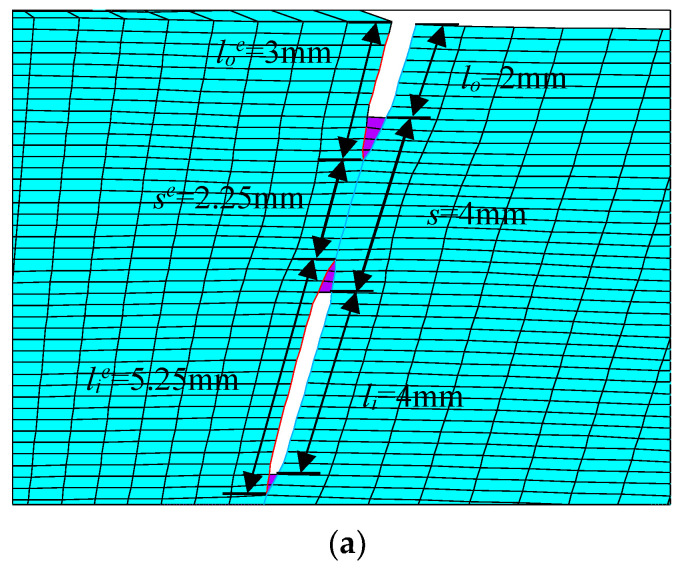

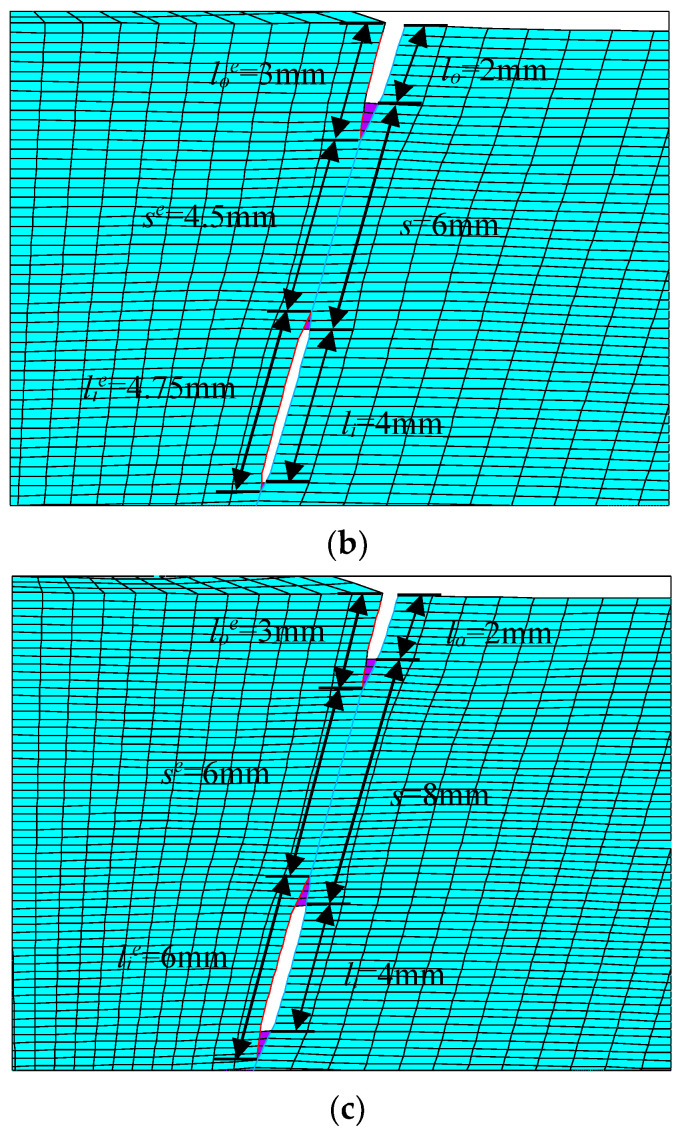
Models of crack tip *T*_1_ extending by 4 finite elements at different spacing: (**a**) *s* = 4 mm; (**b**) *s* = 6 mm; (**c**) *s* = 8 mm.

In order to analyze the influence of crack spacing on crack propagation, finite element models with initial crack spacings *s* = 2 mm, 4 mm, 6 mm, and 8 mm were established, respectively. Figure 15a–c, respectively, show the model diagrams of the crack tip *T*_1_ extending by four finite elements when *s* = 4 mm, *s* = 6 mm, and *s* = 8 mm. When *s* = 2 mm, the model of crack tip *T*_1_ extending by four finite elements is depicted in Figure 12b.

**Figure 16:**
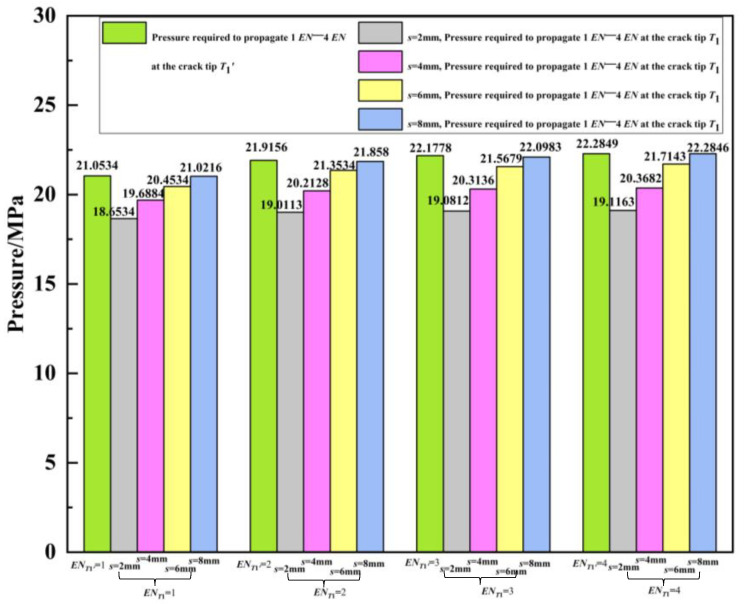
Fluid pressure required for *EN_T_*_1_ = 1, *EN_T_*_1_ = 2, *EN_T_*_1_ = 3, *EN_T_*_1_ = 4.

For different distances, the fluid pressure required is calculated when the double crack tip *T*_1_ extends by one, two, three, and four finite elements. That is, at distances *s* = 2 mm, 4 mm, 6 mm, and 8 mm, respectively, the required fluid pressures are given in Table 2 and Figure 16 when *EN_T_*_1_ = 1, *EN_T_*_1_ = 2, *EN_T_*_1_ = 3, and *EN_T_*_1_ = 4. In Table 2 and Figure 16, to compare the influence of crack spacing on crack interference, the fluid pressures required for the expansion of a single outer wall crack *T*_1_′ extending to one, two, three, and four finite elements are also listed. The interference effects at different spacing from the required pressures can also be found in Table 2 and Figure 16.

**Figure 17:**
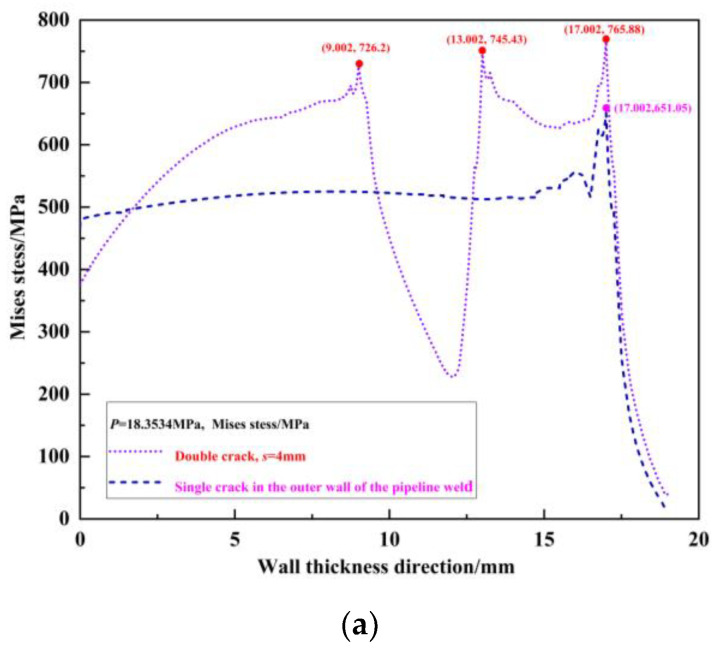

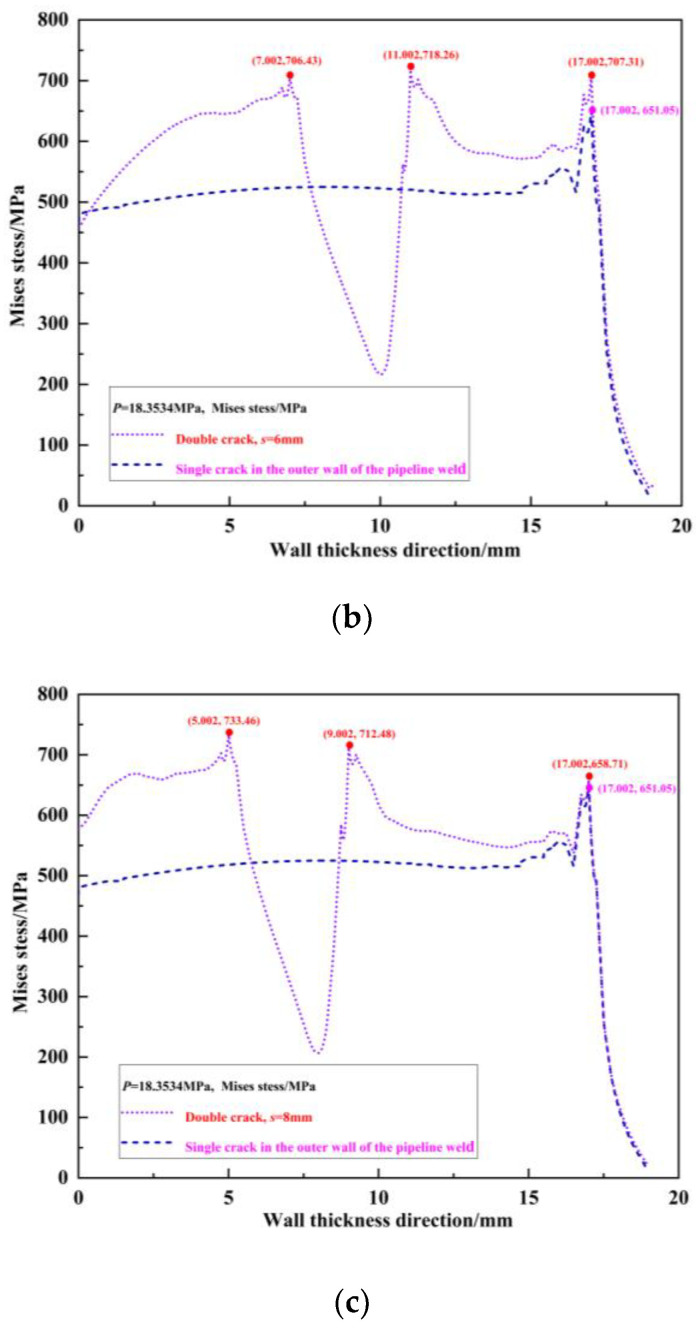
Von Mises stress distribution fields at different spacing under the same pressure load: (**a**) *s* = 4 mm; (**b**) *s* = 6 mm; (**c**) *s* = 8 mm.

When the double-crack distances are s = 2 mm, 4 mm, 6 mm, and 8 mm, respectively, under the same pressure load without any propagation (taking *p* = 18.3534 MPa as an example), the von Mises stress distribution field is extracted, which is shown in Figure 17. To compare the interference effect with various crack spacing, the von Mises stress distribution field of the outer wall single crack is also listed. Figure 17a–c show the von Mises stress distribution field at *s* = 4 mm, *s* = 6 mm, and *s* = 8 mm, respectively. Additionally, the von Mises stress distribution field at *s* = 2 mm can be viewed from Figure 7.

**Figure 18:**
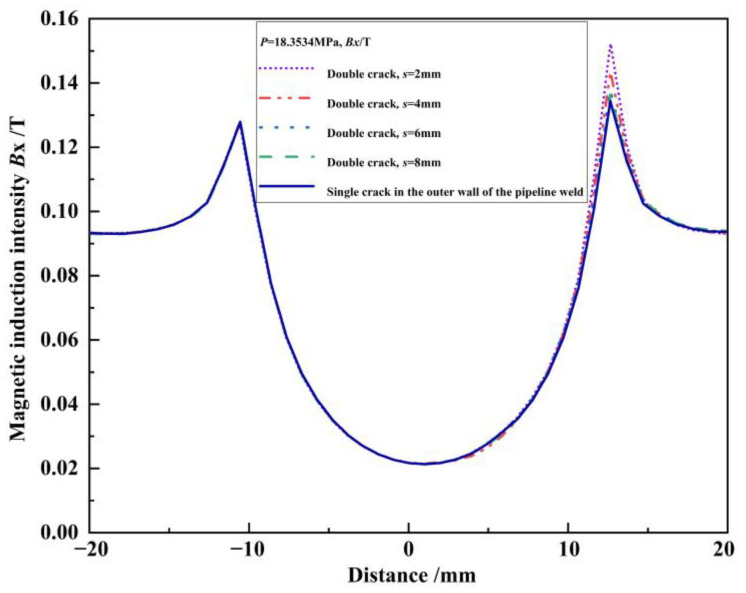
Component curves of magnetic induction intensity at different spacing under the same pressure load.

Under the same pressure load without any propagation (taking *p* = 18.3534 MPa as an example), the magnetic induction intensity component curves of double cracks *s* = 2 mm, 4 mm, 6 mm, and 8 mm are shown in Figure 18. To compare the interference effect with various crack spacing, the magnetic induction intensity component curve of the outer wall single crack is also listed (Figure 8 shows the case for *s* = 2 mm already).

Based on Table 2 and Figure 16, the required fluid pressure increases with the initial spacing *s* of the double cracks. From Figure 7 and Figure 17, under the same pressure load, with the increase in the different initial distances between the double cracks, the peak value of the von Mises stress at the crack decreases. It can be seen from Figure 18 that with the increase in the different initial spacing *s* of the double cracks, the difference between the peak values of the detected magnetic induction intensity components of the double cracks and the values of their symmetrical positions decreases. In order to describe the effect of crack spacing on crack interference more conveniently, the crack interaction scale factors $\gamma_{P}$, $\gamma_{\mathrm{Mises}}$, and $\gamma_{\Delta Bx}$ are introduced. $\;\gamma_{P}\;$ is the ratio of the pressure required for the outer wall single crack to the pressure required for the double cracks when extending by the same number of finite elements; $\gamma_{\mathrm{Mises}}$ is defined as the ratio of the peak value of von Mises stress at the crack tip *T*_1_ of the double cracks to the peak value of the von Mises stress at the crack tip *T*_1_′ of the outer wall single crack under the same pressure load; $\gamma_{\Delta Bx}$ is defined as the ratio of the peak value difference of the magnetic induction intensity component of the double crack to the peak value difference of the magnetic induction intensity component of the single crack in the outer wall under the same pressure load. The expressions are:

(1) $$\gamma_{P}^{o}=\frac{P_{o}}{P_{d}},$$

where *P_o_* is the pressure required for the outer wall single crack, in units of MPa; *P_d_* is the pressure required for the double cracks, in units of MPa.

(2) $$\gamma_{\mathrm{Mises}}^{1}=\frac{{\mathrm{Mises}}_{T1}}{{\mathrm{Mises}}_{{T_{1}}^{\prime }}},$$

where Mises*_T_*_1_ is the peak von Mises stress of the crack tip *T*_1_ of double cracks, in units of MPa; Mises*_T_*_1′_ is the peak von Mises stress of the crack tip *T*_1_′ of the outer wall single crack, in units of MPa.

(3) $$\gamma_{\Delta Bx}^{o}=\frac{\Delta Bx_{d}^{p}}{\Delta Bx_{o}^{p}}\;,$$

where ∆*Bx_d_^p^* represents the peak value difference of the magnetic induction intensity component of the double cracks, in units of T; ∆*Bx_o_^p^* is the peak value difference of the magnetic induction intensity component of the outer wall single crack, in units of T.

Table 3 is obtained from Table 2; Table 4 is obtained from Figure 7 and Figure 17. According to the extraction method of the characteristic quantity ∆*Bx* value in Figure 8, the value of ∆*Bx_d_^p^* when *s* = 2 mm, the value of ∆*Bx_o_^p^* of the outer wall single crack, and the value of ∆*Bx_d_^p^* when s = 4 mm, 6 mm, and 8 mm are extracted from Figure 18 and summarized in Table 5.

It can be seen from Table 3 that when *T*_1_ and *T*_1_′ are extended by the same number of finite elements, the value of $\gamma_{P}^{o}$ decreases as the crack spacing increases. That is, the gap between the pressure required for the outer wall single crack and the pressure required for the double cracks decreases. Taking *EN_T_*_1′_ = 3 and *EN_T_*_1_ = 3 for example, when *s* changes from 2 mm to 4 mm to 6 mm to 8 mm, $\gamma_{P}^{o}$ changes from 1.1623 to 1.0918 to 1.0283 to 1.0036. It can be seen from Table 4 that under the same pressure load, as the crack spacing increases, the value of $\gamma_{\mathrm{Mises}}$ decreases. That is, the difference between the peak von Mises stress of the crack tip *T*_1_ of the double crack and the peak value of the von Mises stress of the crack tip *T*_1_′ of the outer wall single crack decreases. It can be seen from Table 5 that under the same pressure load, as the crack spacing increases, the value of $\gamma_{\Delta Bx}$ decreases. That is, the peak difference between the magnetic induction intensity component of the double crack and the magnetic induction intensity component peak difference of the outer wall single crack decreases. As the crack spacing increases, $\gamma_{P}^{o}$, $\gamma_{\mathrm{Mises}}$, and $\gamma_{\Delta Bx}$ decrease, and the interference effect between the double cracks becomes smaller and smaller. When the crack spacing *s* = 8 mm (double cracks model: the initial length of the outer wall crack *l_o_* = 2 mm, the initial length of the internal crack *l_i_* = 4 mm), the values of $\gamma_{P}^{o}$, $\gamma_{\mathrm{Mises}}$, and $\gamma_{\Delta Bx}$ are close to 1, and the interference effect of the double cracks is negligible. The interference trend of the double cracks gradually weakens with the increasing tip distance and finally tends to the situation of the single crack. Thus, when conducting a safety assessment for multicrack oil and gas pipelines, it is possible to directly simplify the multicrack treatment to the single crack treatment without considering the interaction between cracks.

### 3.3. Effect of Crack Size on Crack Interference

**Figure 19:**
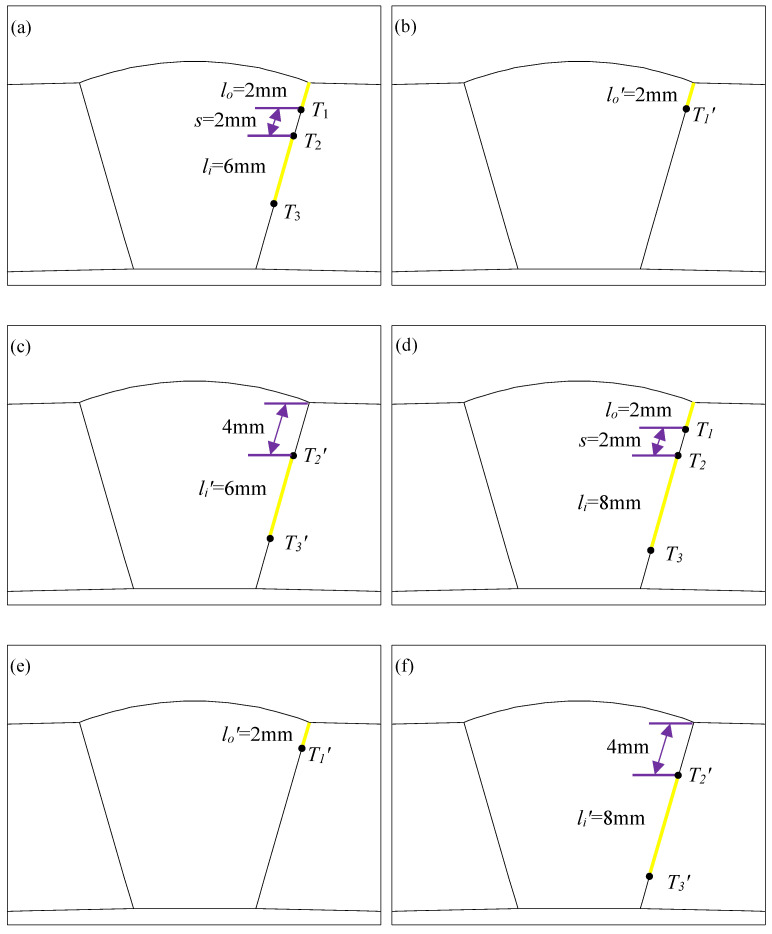
Model diagrams of the first set of examples and the second set of examples: (**a**) double cracks of the first set of examples; (**b**) outer wall single crack of the first set of examples; (**c**) internal single crack of the first set of examples; (**d**) double cracks of the second set of examples; (**e**) outer wall single crack of the second set of examples; (**f**) internal single crack of the second set of examples.

Through the analysis in Section 3.1, compared with the single-crack case, the interference effect between double cracks intensifies the crack propagation process. Three characteristic values are defined as the pressure *P* required for crack propagation, the peak value of von Mises stress, and the difference ∆*Bx* between the peak value of the magnetic induction intensity component at the crack and the value of its symmetrical position. These characteristic values can be used to measure the progress of crack propagation. According to the analysis in Section 3.2, the double cracks’ interference effect weakens as the crack tip distance increases. To further study the interference effect of the unequal-length double cracks, two sets of the above three characteristic quantities are established and extracted. For the first set, the initial crack length in the outer wall is *l_o_* = *l_o_*′= 2 mm, the initial length of the internal crack is *l_i_* = *l_i_*′ = 6 mm, and the initial distance between the crack tips of two unequal-length cracks is *s* = 2 mm. For the second numerical example, the initial length of the outer wall crack is *l_o_* = *l_o_*′ = 2 mm, the initial length of the internal crack is *l_i_* = *l_i_*′ = 8 mm, and the initial distance between the crack tips of two unequal-length cracks is *s* = 2 mm. The model diagrams of the first calculation example are shown in Figure 19a–c, and the model diagrams of the second calculation examples are shown in Figure 19d–f. By comparing the crack interaction scale factors ($\gamma_{P}$, $\gamma_{\mathrm{Mises}}$, $\gamma_{\Delta Bx}$), the interference effect of large-size cracks on small-size cracks and the interference effect of small-size cracks on large-size cracks are studied. Equation (4) is obtained from Formula (1), that is, when extending by the same number of finite elements, the pressure ratio required for the internal single cracks and the double cracks is expressed as:

(4) $$\gamma_{P}^{i}=\frac{P_{i}}{P_{d}},$$

where *P_i_* is the pressure required for the internal single crack, in units of MPa, and *P_d_* is the pressure required for the double cracks, in units of MPa.

The pressure *P* required to extract the characteristic quantity crack propagation process is listed in Table 6 and Table 7. Table 8 is obtained from Formulas (1) and (4) and Table 6 and Table 7.

By comparing the interference scale factors $\gamma_{P}^{o}\;$ and $\;\gamma_{P}^{i}$ in Table 8, it can be seen from the first set of calculation examples (*l_o_* = *l_o_*′ *=* 2 mm, *s* = 2 mm, *l_i_* = *l_i_*′ *=* 6 mm) that when the crack tip extends by one finite element (*EN_T_*_1_ = 1, *EN_T_*_1′_ = 1 or *EN_T_*_2_ = 1, *EN_T_*_2′_ = 1 or *EN_T_*_3_ = 1, *EN_T_*_3′_ = 1), due to the interference of the 6 mm crack on the 2 mm crack, $\gamma_{P}^{o}=\frac{P_{o}}{P_{d}}=1.3197$ (*EN_T_*_1_ = 1, *EN_T_*_1′_ = 1), namely, the extension pressure *P_o_* required for the 2 mm single-crack case is 1.3197 times as large as the expansion pressure *P_d_* required for the 2 mm-and-6 mm double-crack case. Owing to the interference of the 2 mm crack on the 6 mm crack, $\gamma_{P}^{i}=\frac{P_{i}}{P_{d}}=1.1742$ (*EN_T_*_2_ = 1, *EN_T_*_2′_ = 1) and $\gamma_{P}^{i}=\frac{P_{i}}{P_{d}}=1.1805$ (*EN_T_*_3_ = 1, *EN_T_*_3′_ = 1), and the required propagation pressure *P_i_* for the 6 mm single-crack case is 1.1742 times (*EN_T_*_2_ = 1, *EN_T_*_2′_ = 1) and 1.1805 times (*EN_T_*_3_ = 1, *EN_T_*_3′_ = 1) as large as *P_d_* required for the 2 mm-and-6 mm double-crack case. Therefore, the change range of the crack tip propagation pressure of the 2 mm crack under the interference of the 6 mm crack is larger than that of the crack tip propagation pressure in the opposite case. In other words, the proportional factor $\gamma_{P}^{o}>\gamma_{P}^{i}$; the interference effect of 6 mm crack on 2 mm crack is more severe than that of the 2 mm crack on the 6 mm crack. This phenomenon can still be observed when the crack tip extends by two finite elements.

For the second numerical example with *l_o_* = *l_o_*′ *=* 2 mm, *s* = 2 mm, and *l_i_* = *l_i_*′ *=* 8 mm, when the crack tip extends by one finite element (*EN_T_*_1_ = 1, *EN_T_*_1′_ = 1 or *EN_T_*_2_ = 1, *EN_T_*_2′_ = 1 or *EN_T_*_3_ = 1, *EN_T_*_3′_ = 1), $\gamma_{P}^{o}=1.5534$ (*EN_T_*_1_ = 1, *EN_T_*_1′_ = 1), $\gamma_{P}^{i}=1.2213$ (*EN_T_*_2_ = 1, *EN_T_*_2′_ = 1), and $\gamma_{P}^{i}=1.2137\;$(*EN_T_*_3_ = 1, *EN_T_*_3′_ = 1); the interference effect of 8 mm crack on 2 mm crack is more severe than that of 2 mm crack on 8 mm crack. Based on the two numerical examples, it is found that the interference factor ($\gamma_{P}^{o}=1.5534$) of 8 mm crack on the 2 mm crack is $\frac{1.5534}{1.3197}=1.1771$ times as large as the interference factor ($\gamma_{P}^{o}=1.3197$) of the 6 mm crack on the 2 mm crack. The interference factor $\gamma_{P}^{i}=1.2213$ (*EN_T_*_2_ = 1, *EN_T_*_2′_ = 1) and $\gamma_{P}^{i}=1.2137$ (*EN_T_*_3_ = 1, *EN_T_*_3′_ = 1) of the 2 mm crack on 8 mm crack is $\frac{1.2213}{1.1742}=1.0401$ (*EN_T_*_2_ = 1, *EN_T_*_2′_ = 1) times and $\frac{1.2137}{1.1805}=1.0281$ (*EN_T_*_3_ = 1, *EN_T_*_3′_ = 1) times as large as the interference factor $\gamma_{P}^{i}=1.1742$ (*EN_T_*_2_ = 1, *EN_T_*_2′_ = 1) and $\gamma_{P}^{i}=1.1805$ (*EN_T_*_3_ = 1, *EN_T_*_3′_ = 1) of the 2 mm crack on the 6 mm crack. The interference effect of the 2 mm crack on the 6 mm crack and 8 mm crack is not much different. The interference effect of large-size cracks such as 8 mm and 6 mm on small-size cracks such as 2 mm is significantly greater than the influence of the small-size crack such as 2 mm on large-size cracks such as 8 mm and 6 mm. The influence of the 8 mm crack on the 2 mm crack is much greater than that of the 6 mm crack on the 2 mm crack.

In Formula (5), the von Mises stress ratio is defined as, under the same pressure load, the ratio of the von Mises stress peak value at crack tip *T*_2_ of the double crack to that at crack tip *T*_2_′ of the internal single crack. In Formula (6), the von Mises stress ratio is defined as, under the same pressure load, the ratio of the von Mises stress peak value at crack tip *T*_3_ of the double crack to that at crack tip *T*_3_′ of the internal single crack.

(5) $$\gamma_{\mathrm{Mises}}^{2}=\frac{{\mathrm{Mises}}_{T2}}{{\mathrm{Mises}}_{{T_{2}}^{\prime }}}\;,$$

(6) $$\gamma_{\mathrm{Mises}}^{3}=\frac{{\mathrm{Mises}}_{T3}}{{\mathrm{Mises}}_{{T_{3}}^{\prime }}}\;,$$

**Figure 20:**
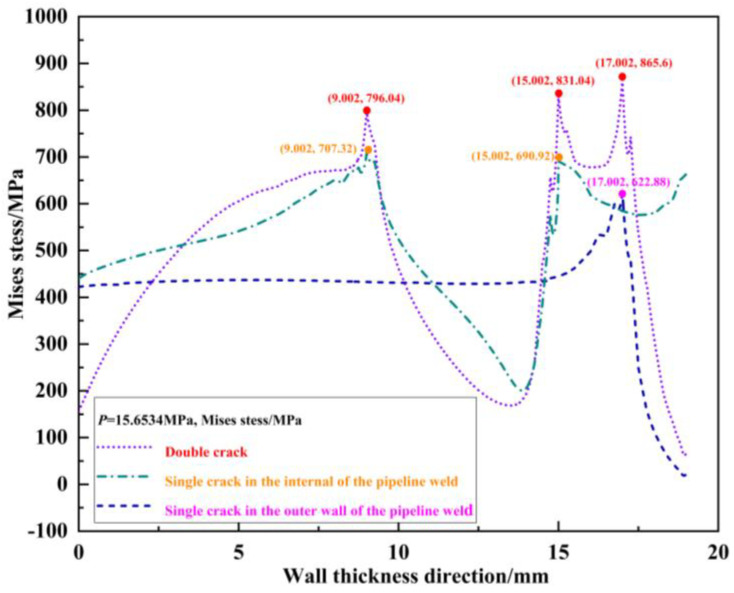
First set of examples.

**Figure 21:**
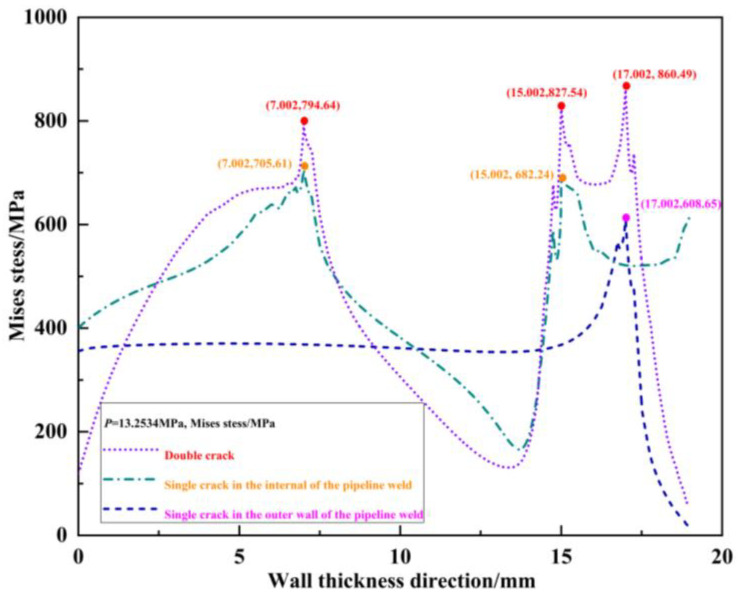
Second set of examples.

Under the same pressure load, *p* = 15.6534 MPa is taken as the first example and *p* = 13.2534 MPa is taken as the second example. The von Mises stress distribution field is shown in Figure 20 and Figure 21. The von Mises stress peak values are extracted from Figure 20 and Figure 21. The results are listed in Table 9 and Table 10 by applying Formulas (2), (5) and (6).

In Table 9 and Table 10, for the first example with *l_o_* = *l_o_*′ *=* 2 mm, *s* = 2 mm, and *l_i_* = *l_i_*′ *=* 6 mm, under the same pressure load *p* = 15.6534 MPa, the interference scale factors including $\gamma_{\mathrm{Mises}}^{1}$, $\gamma_{\mathrm{Mises}}^{2}$, and $\gamma_{\mathrm{Mises}}^{3}$ are compared. Owing to the interference of the 6 mm crack on the 2 mm crack, $\gamma_{\mathrm{Mises}}^{1}=$1.3897, i.e., the von Mises stress peak value at crack tip *T*_1_ for the double-crack case (2 mm and 6 mm, respectively) is 1.3897 times as large as the peak value at the crack tip *T*_1_′ when only the single 2 mm crack exists. Owing to the interference of the 2 mm crack on the 6 mm crack, $\gamma_{\mathrm{Mises}}^{2}=$1.2028 and $\gamma_{\mathrm{Mises}}^{3}=$1.1254, i.e., the von Mises stress peak value at the crack tip *T*_2_ for the double crack case is 1.2028 times as large as the one at crack tip *T*_2_′ when only the single 6 mm crack exists. The von Mises stress peak value at the crack tip *T*_3_ for the double crack case is 1.1254 times as large as the one at crack tip *T*_3_’ when only the single 6 mm crack exists. Therefore, the change amplitude of the von Mises stress peak at the crack tip under the interference of the 6 mm crack to the 2 mm crack is larger than that of the other way around. The scaling factor $\gamma_{\mathrm{Mises}}^{1}>\gamma_{\mathrm{Mises}}^{2}\;\mathrm{and}\;\gamma_{\mathrm{Mises}}^{1}>\gamma_{\mathrm{Mises}}^{3}$, and the interference effect of 6 mm crack to 2 mm crack is stronger than that of the 2 mm crack to 6 mm crack. This phenomenon is more obviously observed in the second example, where *l_o_* = *l_o_*′ *=* 2 mm, *s* = 2 mm, and *l_i_* = *l_i_*′ *=* 8 mm. Under the same pressure load *p* = 13.2534 MPa, $\gamma_{\mathrm{Mises}}^{1}=$1.4138, $\gamma_{\mathrm{Mises}}^{2}=$1.2130, and $\gamma_{\mathrm{Mises}}^{3}=$1.1262, that is, the proportional factor $\gamma_{\mathrm{Mises}}^{1}>\gamma_{\mathrm{Mises}}^{2}\;\mathrm{and}\;\gamma_{\mathrm{Mises}}^{1}>\gamma_{\mathrm{Mises}}^{3}$, and the interference effect of the 8 mm crack to 2 mm crack is more severe than the other way around.

## 4. Conclusions

In the current investigation, a new numerical simulation methodology was developed to study the failure procedure of two interacting cracks in the welding zone of a pipeline. The influence of the space between the two unequal-length cracks as well as the crack sizes were analyzed. Some useful conclusions are drawn as follows:

(1) In pipeline weld joints, cracks with different sizes exist and interfere with each other. It is found that when the unequal length cracks interfere with each other, the cracks are more likely to propagate toward each other, and this trend gradually weakens with the increase in the tip distance, and finally tends to the condition that each crack exists separately.
(2) The crack interaction scale factors ($\gamma_{P}$, $\gamma_{\mathrm{Mises}}$, $\gamma_{\Delta Bx}$) are introduced to gauge the interference intensity. Those scale factors are very user-friendly for practical applications in failure analysis and prevention.
(3) By comparing the degree of mutual interference of two unequal length cracks, the influence of the larger crack on the smaller cracks is greater than that of the smaller one on the larger one.

## Figures and Tables

**Table 1:** Model properties.

Material Parameters	Geometric Dimensioning									Mechanical Properties							
X80	pipeline diameter (mm)						1219			Elastic Moduli *E* (GPa)						180.30	
	pipeline wall thickness (mm)						18.4			Poisson ratio μ						0.3	
	weld width (mm)						22			$\mathrm{Fracture}\;\mathrm{toughness}\;K_{IC}$ $\;(\mathrm{MPa}\sqrt{m})$						115	
	weld reinforcement (mm)						2			Critical strain energy release rate of plane strain model $G_{IC}=\left(1-\mu^{2}\right)\frac{K_{IC}^{2}}{E}$ (N/mm)						66.75	
	Chemical composition of X80 pipeline steel (wt.%)																
	C	Si	Mn	P	S	Cr	Mo	Ni	Nb	V	Ti	Cu	B	Al	N	Ceq	Pcm
	0.045	0.26	1.54	0.013	0.0035	0.14	0.17	0.003	0.050	0.005	0.012	0.15	0.0005	0.012	0.0052	0.37	0.16
Magnetized structure	Dimensions									Materials							
	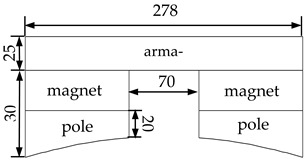									magnet						Nd-Fe-B	
										armature						armco iron	
										pole shoe						armco iron	
Loading	initial internal pressure *P*_s_ (MPa)									1							
	maximum internal pressure *P_e_* (MPa)									30							

**Table 2:** Fluid pressure required for *EN_T_*_1_ = 1, *EN_T_*_1_ = 2, *EN_T_*_1_ = 3, *EN_T_*_1_ = 4.

Single Crack in Outer Wall		Double Cracks with Different Initial Spacing *s*				
*EN_T_*_1′_	*P_o_*/MPa	*EN_T_*_1_	*s* = 2 mm, *P_d_*/MPa	*s* = 4 mm, *P_d_*/MPa	*s* = 6 mm, *P_d_*/MPa	*s* = 8 mm, *P_d_*/MPa
1	21.0534	1	18.6534	19.6884	20.4534	21.0216
2	21.9156	2	19.0113	20.2128	21.3534	21.858
3	22.1778	3	19.0812	20.3136	21.5679	22.0983
4	22.2849	4	19.1163	20.3682	21.7143	22.2846

**Table 3:** $\gamma_{P}^{o}$ under different crack spacing.

*EN*_*T*1_′, *EN*_*T*1_	*s* = 2 mm	*s* = 4 mm	*s* = 6 mm	*s* = 8 mm
1	$\gamma_{P}^{o}=\frac{21.0534}{18.6534}=1.1287$	$\gamma_{P}^{o}=\frac{21.0534}{19.6884}=1.0693$	$\gamma_{P}^{o}=\frac{21.0534}{20.4534}=1.0293$	$\gamma_{P}^{o}=\frac{21.0534}{21.0216}=1.0015$
2	$\gamma_{P}^{o}=\frac{21.9156}{19.0113}=1.1528$	$\gamma_{P}^{o}=\frac{21.9156}{20.2128}=1.0842$	$\gamma_{P}^{o}=\frac{21.9156}{21.3534}=1.0263$	$\gamma_{P}^{o}=\frac{21.9156}{21.858}=1.0026$
3	$\gamma_{P}^{o}=\frac{22.1778}{19.0812}=1.1623$	$\gamma_{P}^{o}=\frac{22.1778}{20.3136}=1.0918$	$\gamma_{P}^{o}=\frac{22.1778}{21.5679}=1.0283$	$\gamma_{P}^{o}=\frac{22.1778}{22.0983}=1.0036$
4	$\gamma_{P}^{o}=\frac{22.2849}{19.1163}=1.1658$	$\gamma_{P}^{o}=\frac{22.2849}{20.3682}=1.0941$	$\gamma_{P}^{o}=\frac{22.2849}{21.7143}=1.0263$	$\gamma_{P}^{o}=\frac{22.2849}{22.2846}=1.0000$

**Table 4:** $\gamma_{\mathrm{Mises}}^{1}$ under different crack spacing.

	Mises*_T_*_1_/MPa	Mises*_T_*_1′_/MPa	$\gamma_{Mises}^{1}=\frac{Mises_{T1}}{Mises_{{T1}^{\prime }}}$
*s* = 2 mm	851.96	651.05	1.3086
*s* = 4 mm	765.88		1.1764
*s* = 6 mm	707.31		1.0864
*s* = 8 mm	658.71		1.0118

**Table 5:** $\gamma_{\Delta Bx}^{o}$ under different crack spacing.

	∆*Bx_d_^p^*/T	∆*Bx_o_^p^*/T	$\gamma_{\Delta Bx}^{o}=\frac{\Delta Bx_{d}^{p}}{\Delta Bx_{o}^{p}}$
*s* = 2 mm	0.0246	0.0065	3.7846
*s* = 4 mm	0.0156		2.400
*s* = 6 mm	0.0102		1.5692
*s* = 8 mm	0.0088		1.3538

**Table 6:** Pressure required for crack propagation process in the first set of examples.

Unequal Length Double Crack		Single Crack in Outer Wall		Internal Single Crack	
	*P_d_*/MPa		*P_o_*/MPa		*P_i_*/MPa
*EN_T_*_1_ = 1	15.9534	*EN_T_*_1′_ = 1	21.0534		
*EN_T_*_2_ = 1	16.1412			*EN_T_*_2′_ = 1	18.9534
*EN_T_*_3_ = 1	16.2588			*EN_T_*_3′_ = 1	19.1943
*EN_T_*_1_ = 2	16.2588	*EN_T_*_1′_ = 2	21.9156		
*EN_T_*_2_ = 2	16.3176			*EN_T_*_2′_ = 2	19.4055
*EN_T_*_3_ = 2	16.347			*EN_T_*_3′_ = 2	19.6164

**Table 7:** Pressure required for crack propagation process in the second set of examples.

Unequal Length Double Crack		Single Crack in Outer Wall		Internal Single Crack	
	*P_d_*/MPa		*P_o_*/MPa		*P_i_*/MPa
*EN_T_*_1_ = 1	13.5534	*EN_T_*_1′_ = 1	21.0534		
*EN_T_*_2_ = 1	13.5534			*EN_T_*_2′_ = 1	16.5534
*EN_T_*_3_ = 1	13.843334			*EN_T_*_3′_ = 1	16.8021
*EN_T_*_1_ = 2	13.7466	*EN_T_*_1′_ = 2	21.9156		
*EN_T_*_2_ = 2	13.7466			*EN_T_*_2′_ = 2	16.8951
*EN_T_*_3_ = 2	13.843337			*EN_T_*_3′_ = 2	17.0805

**Table 8:** Comparison between $\gamma_{P}^{o}$ and $\gamma_{P}^{i}$.

First Set of Examples: *l_o_* = *l_o_*′ *=* 2 mm, *s* = 2 mm, *l_i_* = *l_i_*′ *=* 6 mm			Second Set of Examples: *l_o_* = *l_o_*′ *=* 2 mm, *s* = 2 mm, *l_i_* = *l_i_*′ *=* 8 mm		
*EN*	Effect of Large Size on Small Size	Effect of Small Size on Large Size	*EN*	Effect of Large Size on Small Size	Effect of Small Size on Large Size
*EN_T_*_1_ = 1 *EN_T_*_1′_ = 1	$\gamma_{P}^{o}=\frac{P_{o}}{P_{d}}=\frac{21.0534}{15.9534}=1.3197$		*EN_T_*_1_ = 1 *EN_T_*_1′_ = 1	$\gamma_{P}^{o}=\frac{P_{o}}{P_{d}}=\frac{21.0534}{13.5534}=1.5534$	
*EN_T_*_2_ = 1 *EN_T_*_2′_ = 1		$\gamma_{P}^{i}=\frac{P_{i}}{P_{d}}=\frac{18.9534}{16.1412}=1.1742$	*EN_T_*_2_ = 1 *EN_T_*_2′_ = 1		$\gamma_{P}^{i}=\frac{P_{i}}{P_{d}}=\frac{16.5534}{13.5534}=1.2213$
*EN_T_*_3_ = 1 *EN_T_*_3′_ = 1		$\gamma_{P}^{i}=\frac{P_{i}}{P_{d}}=\frac{19.1943}{16.2588}=1.1805$	*EN_T_*_3_ = 1 *EN_T_*_3′_ = 1		$\gamma_{P}^{i}=\frac{P_{i}}{P_{d}}=\frac{16.8021}{13.843334}=1.2137$
*EN_T_*_1_ = 2 *EN_T_*_1′_ = 2	$\gamma_{P}^{o}=\frac{P_{o}}{P_{d}}=\frac{21.9156}{16.2588}=1.3479$		*EN_T_*_1_ = 2 *EN_T_*_1′_ = 2	$\gamma_{P}^{o}=\frac{P_{o}}{P_{d}}=\frac{21.9156}{13.7466}=1.5943$	
*EN_T_*_2_ = 2 *EN_T_*_2′_ = 2		$\gamma_{P}^{i}=\frac{P_{i}}{P_{d}}=\frac{19.4055}{16.3176}=1.1892$	*EN_T_*_2_ = 2 *EN_T_*_2′_ = 2		$\gamma_{P}^{i}=\frac{P_{i}}{P_{d}}=\frac{16.8951}{13.7466}=1.2290$
*EN_T_*_3_ = 2 *EN_T_*_3′_ = 2		$\gamma_{P}^{i}=\frac{P_{i}}{P_{d}}=\frac{19.6164}{16.347}=1.2$	*EN_T_*_3_ = 2 *EN_T_*_3′_ = 2		$\gamma_{P}^{i}=\frac{P_{i}}{P_{d}}=\frac{17.0805}{13.843337}=1.2338$

**Table 9:** $\gamma_{\mathrm{Mises}}$ of first set of examples (*p* = 15.6534 MPa).

*T*_1_ and *T*_1_′		*T*_2_ and *T*_2_′		*T*_3_ and *T*_3_′	
Mises*_T_*_1_/MPa	865.60	Mises*_T_*_2_/MPa	831.04	Mises*_T_*_3_/MPa	796.04
Mises*_T_*_1′_/MPa	622.88	Mises*_T_*_2′_/MPa	690.92	Mises*_T_*_3′_/MPa	707.32
$\gamma_{\mathrm{Mises}}^{1}=\frac{{\mathrm{Mises}}_{T1}}{{\mathrm{Mises}}_{{T1}^{\prime }}}$	1.3897	$\gamma_{\mathrm{Mises}}^{2}=\frac{{\mathrm{Mises}}_{T2}}{{\mathrm{Mises}}_{{T2}^{\prime }}}$	1.2028	$\gamma_{\mathrm{Mises}}^{3}=\frac{{\mathrm{Mises}}_{T3}}{{\mathrm{Mises}}_{{T3}^{\prime }}}$	1.1254

**Table 10:** $\gamma_{\mathrm{Mises}}$ of second set of examples (*p* = 13.2534 MPa).

*T*_1_ and *T*_1_′		*T*_2_ and *T*_2_′		*T*_3_ and *T*_3_′	
Mises*_T_*_1_/MPa	860.49	Mises*_T_*_2_/MPa	827.54	Mises*_T_*_3_/MPa	794.64
Mises*_T_*_1′_/MPa	608.65	Mises*_T_*_2′_/MPa	682.24	Mises*_T_*_3′_/MPa	705.61
$\gamma_{\mathrm{Mises}}^{1}=\frac{{\mathrm{Mises}}_{T1}}{{\mathrm{Mises}}_{{T1}^{\prime }}}$	1.4138	$\gamma_{\mathrm{Mises}}^{2}=\frac{{\mathrm{Mises}}_{T2}}{{\mathrm{Mises}}_{{T2}^{\prime }}}$	1.2130	$\gamma_{\mathrm{Mises}}^{3}=\frac{{\mathrm{Mises}}_{T3}}{{\mathrm{Mises}}_{{T3}^{\prime }}}$	1.1262

## Data Availability

Not applicable.
